# A Case of Hydrocele Cured by Electro Magnetism

**Published:** 1846-06

**Authors:** Thomas L. Ogier


*A case of Hydrocele cured by Electro Magnetism.* By THOMAS L. OGIER.—An old gentleman, whom I was called to attend in November, 1845, for an enlargement of the prostate gland and paralysis of the bladder, had a hydrocele of the right testicle, which caused him great inconvenience, and which he was very anxious I should operate upon. His health at that time was such as to render an operation improper, and I therefore advised him to wait until it should improve, intending then to operate by injection ; or if his health did not warrant this, to give him temporary relief by simply puncturing the tumor and letting out the fluid. For the affection of the bladder, it was thought proper to apply the electro-magnetic battery, and pass the shocks from the lower part of the spine through the bladder, in all directions. Whilst making these applications, I felt desirous to know what would be the effect of passing the electric fluid through the hydrocele, and therefore determined to try it. The wires were applied to the tumor and the electricity allowed to pass through it in every direction ; this application was made every day for a fortnight, and each application was continued from six to eight or ten minutes. In about ten days the swelling was increased, the testicle itself became enlarged, and painful when pressed, the scrotum was red or slightly œdematous, and the whole tumor assumed very much the appearance of a hydrocele two or three days after the operation by injection. It remained in this condition two or three days, and then gradually subsided ; and three weeks after, the parts became of the natural size. It is now more than two months since the application of the battery. The testicle remains in its normal state, the hydrocele evidently radically cured. I am not aware that hydrocele has ever before been treated by the above method. As it produces no constitutional irritation, and is of such easy application, would it not be expedient in recent cases, where there is not much thickening of the tunica vaginalis, and also in hydrocele occurring in old debilitated subjects? —*Ibid.*

